# The Effect and Dose-Response of Functional Electrical Stimulation Cycling Training on Spasticity in Individuals With Spinal Cord Injury: A Systematic Review With Meta-Analysis

**DOI:** 10.3389/fphys.2021.756200

**Published:** 2021-11-19

**Authors:** Chia-Ying Fang, Angela Shin-Yu Lien, Jia-Ling Tsai, Hsiao-Chu Yang, Hsiao-Lung Chan, Rou-Shayn Chen, Ya-Ju Chang

**Affiliations:** ^1^School of Physical Therapy and Graduate Institute of Rehabilitation Science, College of Medicine, Chang Gung University, Taoyuan, Taiwan; ^2^School of Nursing, College of Medicine, Chang Gung University, Taoyuan, Taiwan; ^3^Healthy Aging Research Center, Chang Gung University, Taoyuan, Taiwan; ^4^Division of Endocrinology and Metabolism, Department of Internal Medicine, Chang Gung Memorial Hospital, Taoyuan, Taiwan; ^5^Department of Electrical Engineering, College of Engineering, Chang Gung University, Taoyuan, Taiwan; ^6^Neuroscience Research Center, Chang Gung Memorial Hospital, Taoyuan, Taiwan; ^7^Department of Neurology, Chang Gung Memorial Hospital, Taoyuan, Taiwan; ^8^School of Medicine, College of Medicine, Chang Gung University, Taoyuan, Taiwan

**Keywords:** spinal cord injury, functional electrical stimulation, cycling, spasticity, dose-response

## Abstract

**Background:** To investigate the effect and dose-response of functional electrical stimulation cycling (FES-cycling) training on spasticity in the individuals with spinal cord injury (SCI).

**Method:** Five electronic databases [PubMed, Scopus, Medline (Proquest), Embase, and Cochrane Central Register of Controlled Trials (CENTRAL)] were searched before September 2021. The human trials and studies of English language were only included. Two authors independently reviewed and extracted the searched studies. The primary outcome measure was spasticity assessed by Modified Ashworth Scale or Ashworth Scale for lower limbs. The secondary outcome measures were walking abilities, such as 6 Min Walk Test (6MWT), Timed Up and Go (TUG), and lower limbs muscle strength (LEMS). A subgroup analysis was performed to investigate the efficacious threshold number of training sessions. A meta-regression analysis was used to examine the linear relationship between the training sessions and the effect on spasticity.

**Results:** A total of 764 studies were identified. After screening, 12 selected studies were used for the qualitative synthesis, in which eight of them were quantitatively analyzed. Eight studies included ninety-nine subjects in total with SCI (male: female = 83:16). The time since injury was from less than 4 weeks to 17 years. The age ranged from 20 to 67 years. American Spinal Injury Association (ASIA) impairment level of the number of participants was 59 for ASIA A, 11 for ASIA B, 18 for ASIA C, and 11 for ASIA D. There were 43 subjects with tetraplegia and 56 subjects with paraplegia. Spasticity decreased significantly (95% *CI* = − 1.538 to − 0.182, *p* = 0.013) in favor of FES-cycling training. The walking ability and LEMS also improved significantly in favor of FES-cycling training. The subgroup analysis showed that spasticity decreased significantly only in more than 20 training sessions (95% *CI* = − 1.749 to − 0.149, *p* = 0.020). The meta-regression analysis showed training sessions and spasticity were not significantly associated (coefficient = − 0.0025, SE = 0.0129, *p* = 0.849, *R*^2^ analog = 0.37).

**Conclusion:** Functional electrical stimulation-cycling training can improve spasticity, walking ability, and the strength of the lower limbs in the individuals with SCI. The number of training sessions is not linearly related to the decrease of spasticity. Twenty sessions of FES-cycling training are required to obtain the efficacy to decrease spasticity.

## Introduction

According to the National Spinal Cord Injury Statistics, the annual incidence of spinal cord injury (SCI) is 17,730 in the United States of America (National Spinal Cord Injury Statistical Center [NSCISC], 2017). SCI causes permanent changes in strength, sensation, and autonomic dysfunction below the level of the injury. SCI has a profound impact on the life of an individual. Exercise is recommended not only for able-bodied persons but also for people with SCI. Recent rehabilitation strategies have focused on the early repetitive task-oriented approaches to facilitate central nervous system plasticity in people with SCI (Fouad and Tetzlaff, 2012). Improving the functional activities of this population is vital wherein locomotor training using functional electrical stimulation cycling (FES-cycling) has been provided in clinical rehabilitation.

Spasticity is an upper motor neuron syndrome that occurred in 65% of individuals with SCI at discharge (Holtz et al., 2017). Severe spasticity deteriorates motor functions and is related to the presence of pain, lower quality of life, and daily activities (Milinis et al., 2015; McKay et al., 2018). The non-invasive managements of spasticity include medication, e.g., baclofen (Lin and Chay, 2018), neuromuscular electrical stimulation (NMES) (Khan et al., 2017), stretching, heat, cold, vibration, and positioning (Naro et al., 2017). However, between 25 and 70% of patients with oral baclofen reported adverse effects, such as muscle weakness, somnolence, nausea, and dizziness (Ertzgaard et al., 2017). The effect of passive stretching on spasticity was short-term (Chang et al., 2007). Therefore, it is essential to find an appropriate approach to alleviate spasticity, especially for those who response poorly to medication. Several methods are used to quantify spasticity level, in these, the Ashworth Scale (AS) and Modified Ashworth Scales (MAS) are the most common methods to assess spasticity clinically (Skold et al., 1998). The inter- and intra-rater reliability of MAS was moderate to good, influenced by the type of study design, upper or lower limbs, and the number of raters (Meseguer-Henarejos et al., 2018). A previous study revealed that the prevalence of spasticity measured by MAS was correlated with spastic muscle electromyogram (EMG) (Skold et al., 1998).

In addition to spasticity, walking ability is an important clinical goal of people with SCI, especially for people with incomplete injury. The walking ability is influenced by various factors, such as lower extremity muscle strength, endurance, balance, and spasticity levels. Six Minute Walk Test (6MWT), Timed Up and Go (TUG), and lower limbs muscle strength (LEMS) were commonly employed to assess walking function and lower extremity muscle strength in SCI (van Hedel et al., 2005). These tests were reported to correlate with endurance (Jackson et al., 2008) and balance (Mathias et al., 1986), respectively.

Functional electrical stimulation-cycling is a safe and efficient rehabilitation strategy for SCI. In that, the surface electrodes are applied on quadriceps, hamstrings, and gluteus to activate the paralyzed muscles sequentially to induce pedaling movement. FES-cycling has been observed with several benefits, such as increasing muscle strength (Sadowsky et al., 2013; Kuhn et al., 2014), cardiopulmonary fitness (Davis et al., 2008), bone density (Frotzler et al., 2008, 2009), and decreasing spasticity (Sadowsky et al., 2013; Mazzoleni et al., 2017; Naro et al., 2017; Popovic-Maneski et al., 2018). Sadowsky et al. (2013) reported that the motor score significantly improved after 45–60 min, 3 sessions per week, average 29.5 months of FES-cycling training in people with chronic SCI. Muscle atrophy was prevented after FES-cycling training for 3 months in acute phase of SCI (Baldi et al., 1998). Improvement in peak VO_2_ was observed after a minimum of 24 training sessions, 30 min over a 19-week period of FES-cycling training in people with SCI (Hooker et al., 1995).

The advantage of FES-cycling was making exercise possible for the individuals with paralysis (Peng et al., 2011). The FES-cycling includes FES portion and cycling portion. The past studies revealed one or multiple sessions of FES-only decreased spasticity (Naro et al., 2017; Sivaramakrishnan et al., 2018), however, some research reported no effect on spasticity in people with SCI (Kapadia et al., 2014; Thomaz et al., 2019). The effects of FES-cycling training on spasticity are still controversial (Peng et al., 2011). Several studies showed FES-cycling training could reduce spasticity in people with SCI (Krause et al., 2008; Kuhn et al., 2014; Mazzoleni et al., 2017; Naro et al., 2017; Popovic-Maneski et al., 2018), stroke (Lo et al., 2009), and multiple sclerosis (Motl et al., 2006). However, Arnold et al. (1992) found that FES-cycling training 30 min per session for 3 months improved cardiovascular fitness and thigh girths, but spasticity became intense in the individuals with SCI. Some studies showed no effect of FES-cycling training on spasticity in the patients with SCI (Sköld et al., 2002; Mazzoleni et al., 2013; Ralston et al., 2013). These controversial findings may be due to small sample sizes, heterogeneity of the studied populations, different training protocols, and the different assessment tools used. The limitation of single study is small sample size. Thus, pooling more studies to investigate more accurate result on the effect is necessary. In additions, the controversial results could due to the dosage difference. The optimal dose-response of training sessions related to the effect of FES-cycling training on spasticity needed to be studied as well.

The high-quality evidence, such as meta-analysis, of FES-cycling on reduction of spasticity is lacking. Barbosa et al. (2021) performed a meta-analysis of physical therapy intervention on spasticity and found inconclusive effect. This might be due to the heterogeneity of the included studies in which only one study used FES-cycling. Recently, the systemic review from Alashram et al. (2020) suggested that the FES-cycling intervention may reduce the lower extremities spasticity in the patients with various injury levels of SCI. However, the meta-analysis was not performed in the study of Alashram to confirm the effect. In additions, spasticity occurs as an adaptation process of time post injury, and the sessions of intervention are critical wherein the dose response should be considered. Therefore, the purpose of this study is to use systemic review with meta-analysis to investigate the effect of FES-cycling training on spasticity in individuals with SCI, with dose-response, i.e., training sessions, analyzed.

## Methods

The results from relevant studies were integrated following the systematic review and meta-analysis guidelines outlined in the Preferred Reporting Items for Systematic Review and Meta-Analysis statement (Moher et al., 2015). This meta-analysis was registered *a priori* with PROSPERO on Feb 11, 2021 (CRD42021230762)^1^.

### Types of Participants

The current meta-analysis included only adults with SCI, regardless of traumatic or non-traumatic injury, the time since injury, and gender.

### Types of Interventions

Functional electrical stimulation combined with active cycling training for lower limbs in the individuals with SCI was included. FES-cycling combined with other modality, such as resistance training, overground walking, and locomotor training, was excluded to decrease the heterogeneity and avoid confounding factors.

### Outcome Measures

The primary outcome measure was spasticity assessed by MAS or AS for lower limbs. The secondary outcome measures were walking abilities, such as 6MWT, TUG, and LEMS. The 6MWT measured the walking distance in 6 min. TUG assessed the time that a subject took to rise from a chair, walk 3 m, turn around, walk back to the chair, and sit down. LEMS assessed motor score for lower limbs according to American Spinal Injury Association (ASIA) standard.

### Type of Studies

Because there were few randomized control trial (RCT) studies, both RCT and non-RCT studies were included in this meta-analysis.

### Searching Criteria

The searching criteria were limited to the human studies and English language.

### Data Sources

Five electronic databases [PubMed, Scopus, Medline (Proquest), Embase, and Cochrane Central Register of Controlled Trials (CENTRAL)] before September 2021 were searched. Medical Subject Heading terms were combined with keywords to search. Searching key words included cycling, functional electrical stimulation, FES, spinal cord injury and spasticity in title, abstract, and keywords. Supplementary Appendix 1 shows the combinations used. The hand searching from references lists for relevant studies was also done.

Two authors reviewed and screened the bibliographies of articles and abstracts related to the FES-cycling training in the individuals with SCI independently.

### Study Selection

Two authors independently searched and screened the titles, abstracts, and literatures to identify the potentially relevant studies. Then, full texts of relevant studies were obtained and assessed to determine whether the articles met the inclusion criteria. Any disagreement was discussed and solved with a third author to reach consensus in every relevant detail.

### Data Extraction and Management

Two authors extracted data independently from the included studies and filled into an extraction form. The following data were extracted: (1) authors; (2) year of publication; (3) study design; (4) inclusion/exclusion criteria; (5) subject demographics (age, gender, number of subjects, level of lesion, classification of ASIA, and time since injury); (6) intervention; and (7) outcome measures.

The data measured at the beginning (baseline) and at the end of interventions were extracted for meta-analysis. We did not analyze the measurements taken during interventions or at follow-up due to inconsistent measuring time points used across different studies. The studies were excluded if the primary outcome measures were missing or not measured. Of these included studies, two met the inclusion criteria from all searching sources but had only one-session intervention, were also included and analyzed separately.

### Quality Assessment

The methodological quality of the selected RCT studies was independently assessed by the two authors using the Cochrane risk of bias assessment tool (Higgins and Altman, 2008). For the assessment of the quality of the selected clinical trials, the Newcastle Ottawa Scale (Wells et al., 2000; Stang, 2010) was employed and done by two authors independently. Any disagreement was resolved through discussion and consensus with a third author. The publication bias was analyzed by using the funnel plots.

### Statistical Analysis

As only one RCT was included, all the included RCT and non-RCT studies were grouped for analysis, except two studies. These two studies were analyzed separately due to only one-session performance of FES-cycling. The scores of MAS and AS of lower limbs were pooled for the analysis. Mean differences and 95% *CI* were calculated for each primary and secondary outcome measure. The pooled mean difference estimates were calculated according to the random effect models if heterogeneous was significant. The fixed effect models were used to calculate the pooled mean difference estimates if no heterogeneous occurred. Because the dose of number of training sessions was varied across the studies, the effectiveness of spasticity was unknown. In such condition, a subgroup analysis was performed according to the training sessions. As a result of 20 electrical stimulation training sessions decreasing spasticity in stroke (Stein et al., 2015; Nakipoğlu Yuzer et al., 2017), 20 training sessions were set as the cut-point, whereas below 20 training sessions were classified as subgroup 1, and the others were classified as subgroup 2. A meta-regression analysis was also analyzed to examine the linear relationship between the training sessions and spasticity. The random effect model was chosen in meta-regression.

Comprehensive Meta-Analysis Software (Version 3) was used to analyze the data. A *p*-value less than 0.05 was considered as significant.

## Results

### Studies Included

**FIGURE 1:**
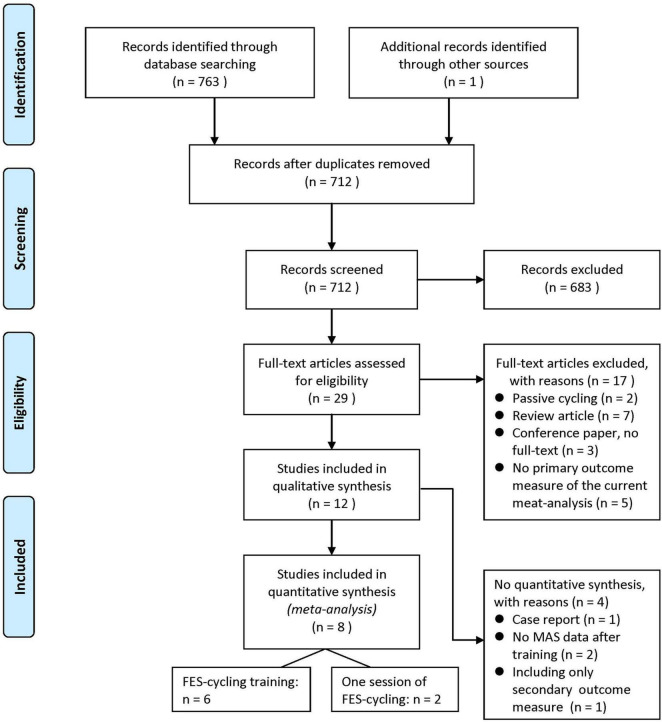
Flow diagram of the study selection process.

A total of 763 studies were identified from the electronic databases and one additional study from hand searching from references lists of available articles. After removing 52 repeated articles, the authors screened and assessed full text for eligibility. There were 12 studies for qualitative synthesis, such as one RCT study and 11 non-RCT studies. Eight of them were quantitatively analyzed for meta-analysis (Sköld et al., 2002; Krause et al., 2008; Szecsi and Schiller, 2009; Mazzoleni et al., 2013, 2017; Ralston et al., 2013; Kuhn et al., 2014; Yas̨ar et al., 2015), and four studies were excluded due to reasons, such as case report (*n* = 1) (Fattal et al., 2018), no MAS result of post-test (*n* = 2) (Reichenfelser et al., 2012; Popovic-Maneski et al., 2018), and only LEMS data included (*n* = 1) (Sadowsky et al., 2013). The flowchart is shown as Figure 1. The characteristics of all the included studies are shown in Table 1.

**TABLE 1:** The characteristics of the included studies.

Study	Research design	Participants	Intervention	Outcome measures	Quality score
Ralston et al. (2013)	Randomized cross-over trial	*n* = 14 ASIA A, B, C Level of injury: C4 to T10	Four times a week, 30–45 min of FES driven leg cycling (RT300 cycle) within a 1-h session 2 weeks FES-cycling, and 2 weeks control phase	AS (quadriceps, hamstrings, plantarflexor, and hip adductor) PRISM Lower limb swelling Urine output Global Impression of Change Scale	5
Fattal et al. (2018)	Case report	*n* = 1 ASIA A Level of injury: T3	6 month period of isometric stimulation of the sublesional muscles and the 6 month period of FES cycling, on a stationary bike (Berkelbike PRO^®^), then on a competition bike (Ice Trike Adventure^®^) Two to three 30-min sessions per week	VAS MAS (hip add., knee flex, ext, ankle plantarflex.) Perceived effort (Borg scale) Thigh circumference Body composition	6
Kuhn et al. (2014)	Prospective clinical cohort study	*n* = 30 ASIA A, B, C, D Level of injury: below C4	20-min FES-cycling program (MOTOmed Viva 2) 2–3 days per week for 4 weeks	MAS (hip abd., add., knee ext., flex., and dorsal ext. or plantarflex.) Thigh circumference Muscle size MMT/WISCI II/TUG/6MWT	6
Mazzoleni et al. (2017)	Single group	*n* = 7 ASIA A Level of injury: T4 to T12	(1)*n* = 20 sessions, 3 sessions/wk, FES-cycling system (Pegaso, Biotech Srl, Italy) (2)*n* = 20 sessions, 3 sessions/wk, overground robotic exoskeleton (Ekso GT, Ekso Bionics, United States)	MAS PSFS SCIM Subjective spasticity and pain [0–10 points Numerical Rating Scale (NRS)] pain and quality of life (QoL) (ISCI) 10MWT/6MWT/TUG	6
Mazzoleni et al. (2013)	Single group	*n* = 5 ASIA A, B, C Level of injury: C7, T10, T12	FES-cycling system (Pegaso, Biotech Srl, Italy) 20 sessions, three sessions per week for 7 weeks The first session was addressed to familiarization, the duration of the second session was 15 min, from the third to the twentieth session 5 min were incrementally added, till to 30 min	SCIM MAS 4-point Spasms Scale Evaluation of muscle area through measurement of thigh circumference at 5, 10, and 15 cm from the knee cap upper limit	6
Popovic-Maneski et al. (2018)	Two groups	Healthy controls, *n* = 6 SCI, *n* = 9 ASIA B Level of injury: C7, T10, T12	F group: RT300 FES cycling system (Restorative Therapies Inc., United States), 6 months three times weekly for 1 h periods C group: lower-limb mobilization therapy	Pendulum test AS (knee joint)	7
Reichenfelser et al. (2012)	Two groups	iSCI, *n* = 23 healthy controls, *n* = 13 ASIA B, C, D Level of injury: C4 to L1	Commercially available tricycle (AnthroTech Leichtfahrzeugtechnik GmbH, Eckental, Germany) that was adapted 33.6 ± 6.1 min, 3 times a week, 2 months	MAS (knee joint)	7
Sadowsky et al. (2013)	Two groups	FES-cycling, *n* = 25 Control, *n* = 20 ASIA A, B, C Level of injury: C1 to L5	ERGYS2 FES cycle ergometers (Therapeutic Alliances Inc., Fairborn, OH, United States) 45–60 min, 3 sessions per week, average 29.5 months Controls received non-center based passive stretching with no active physical therapy	Spasticity: isokinetic dynamometer, resistance torque (knee and ankle) ASIA motor, sensory score Muscle, fat, bone density Blood count, metabolic profile, and fasting lipid profile SF-36 FIM	7
Sköld et al. (2002)	Two groups	Cycling, *n* = 8 Control, *n* = 7 ASIA A, B Level of injury: C1 to T1	FES bicycle (ERGYS I Clinical Rehabilitation System, Therapeutic Technology, Tampa, United States) 30-min sessions 3 times weekly for 6 months	Body composition MAS (rectus femoris and the lateral biceps femoris muscles) isokinetic Kin-Com: resistive torque VAS (spasticity)	7
Yas̨ar et al. (2015)	Prospective single-arm experimental design	*n* = 10 ASIA C, D Level of injury: C4 to T12	FES cycling system (RT 300-SLSA; Restorative Therapies, Baltimore, MD, United States) One hour session, 3 times a week for 16 weeks, follow up 6 months	Total motor score from the standardized ASIA clinical exam FIM MAS (both knees) Three-dimensional gait analysis Oxygen consumption during walking	6
Krause et al. (2008)	Crossover study	*n* = 5 ASIA A Level of injury: T3 to T7	Active session with FES and the passive movement session, 60–100 min	MAS (knee extensors) Pendulum test	5
Szecsi and Schiller (2009)	Single group	*n* = 13 ASIA A Level of injury: C7 to T12	1. Isometric torque generation using LFRP and MFAC stimulation 2. Ergometry using LFRP stimulation 3. Ergometry using MFAC stimulation Ergometry (Reck-Technik Ltd and Co, 88422 Betzenweiler, Germany)	MAS (knee joint) Isometric torque	5

*AS, Ashworth scale; PRISM, Patient Reported Impact of Spasticity Measure; VAS, Visual Analog Scale; MAS, modified Ashworth scale; WISCI, Walking Index for Spinal Cord Injury; TUG, Timed up and go test; 6MWT, 6-min walk test; PSFS, Penn Spasm Frequency Scale; SCIM, Spinal Cord Independence Measure; ISCI, International Spinal Cord Injury Pain Data Set; 10MWT, 10-m walking test; SF-36, Short Form 36; FIM, Functional Independence Measure; MFAC, middle frequency alternating current; LFRP, low frequency rectangular pulse.*

### Excluded Studies

After screening, 683 studies were eliminated and 29 full-text articles were assessed for eligibility. The reasons for exclusion were as follows: passive cycling, review articles, no full text, and no assessing primary outcome measure of the current meta-analysis.

### Study Location

Among 12 included studies, 2 trials were done in the United States (Sköld et al., 2002; Sadowsky et al., 2013), 2 in Italy (Mazzoleni et al., 2013, 2017), 3 in Germany (Krause et al., 2008; Szecsi and Schiller, 2009; Kuhn et al., 2014), others in Australia (Ralston et al., 2013), Austria (Reichenfelser et al., 2012), France (Fattal et al., 2018), Serbia (Popovic-Maneski et al., 2018), and Turkey (Yas̨ar et al., 2015).

### Study Participants

The eight studies for quantitative analysis included 99 subjects with SCI (male: female = 83:16). The age of the participants ranged from 20 to 67 years. The time since injury was below 4 weeks to 17 years. ASIA level of the number of participants was 59 for ASIA A, 11 for ASIA B, 18 for ASIA C, and 11 for ASIA D. There were 43 subjects with tetraplegia and 56 subjects with paraplegia.

The four studies which were excluded for the quantitative analysis comprised 78 participants with SCI and 19 healthy controls (male: female = 80:17). The age of the participants ranged from 22 to 62 years. The time since injury was from 1 month to 21 years. ASIA level of the number of participants was 32 for ASIA A, 22 for ASIA B, 15 for ASIA C, and 9 for ASIA D. There were 43 subjects with tetraplegia and 35 subjects with paraplegia.

### Interventions

The frequency of FES was from 20 to 100 Hz and pulse width was from 50 to 600 μs in all the included studies. The FES-cycling training protocol was ranged from 20 min to 1 h per session, 2 to 4 sessions per week, and 2 to 118 weeks in total.

### Risk of Bias of the Included Studies

Tables 2, 3 summarize the risk of bias judgments related to one RCT and 11 non-RCT studies. The RCT study (Ralston et al., 2013) and the nine non-RCT studies had good quality, and the two non-RCT studies (Krause et al., 2008; Szecsi and Schiller, 2009) comprising one session of FES-cycling were of moderate quality.

**TABLE 2:** Risk of bias summary for assessing the quality of included randomized control trial (RCT) study.

	Random sequence generation (selection bias)	Allocation concealment (selection bias)	Blinding of participants and personnel (performance bias)	Blinding of outcome assessment (detection bias)	Incomplete outcome data (attrition bias)	Selective reporting (reporting bias)	Other bias

Ralston et al. (2013)	**+**	**+**	**−**	**+**	**+**	**+**	**?**

*The judgments of reviewers about each risk of bias for each included study. +, low risk of bias; −, high risk of bias; ?, unclear risk of bias.*

**TABLE 3:** Newcastle-Ottawa Scale for assessing the quality of included non-RCT studies.

		Selection				Comparability		Outcome			
Study ID	Year	S1	S2	S3	S4	C1	C2	O1	O2	O3	No. of star
Fattal et al.	2018	★		★	★			★	★	★	6
Kuhn et al.	2014	★		★	★			★	★	★	6
Mazzoleni et al.	2017	★		★	★			★	★	★	6
Mazzoleni et al.	2013	★		★	★			★	★	★	6
Popovic-Maneski et al.	2018	★	★	★	★			★	★	★	7
Reichenfelser et al.	2012	★	★	★	★			★	★	★	7
Sadowsky et al.	2013	★	★	★	★			★	★	★	7
Sköld et al.	2002	★	★	★	★			★	★	★	7
Yas̨ar et al.	2015	★		★	★			★	★	★	6
Krause et al.	2008	★		★	★			★		★	5
Szecsi and Schiller	2009	★		★	★			★		★	5

### Effects of the Interventions

#### Effects on Spasticity

**FIGURE 2:**
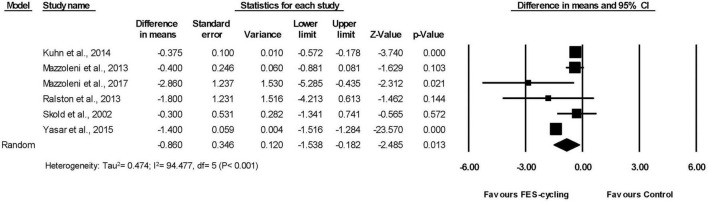
A forest plot of spasticity after functional electrical stimulation (FES)-cycling training.

**FIGURE 3:**
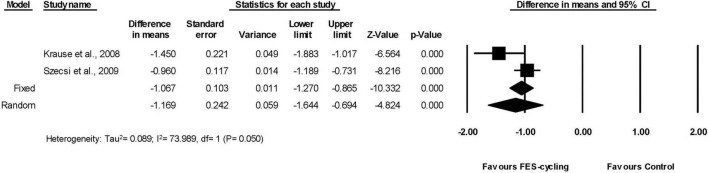
A forest plot of spasticity after one-session of FES-cycling.

Six included studies (Sköld et al., 2002; Mazzoleni et al., 2013, 2017; Ralston et al., 2013; Kuhn et al., 2014; Yas̨ar et al., 2015), which had multiple intervention sessions measured the spasticity of lower limbs by MAS. The results showed that the MAS score decreased significantly compared with the pre-training status (95% *CI* = −1.538 to −0.182, *p* = 0.013) in favor of FES-cycling training. The pooled mean difference (random effect model) was −0.86 for these six studies (Figure 2). The other two studies (Krause et al., 2008; Szecsi and Schiller, 2009), which had only one intervention session, also showed that the MAS score decreased significantly (95% *CI* = −1.644 to −0.694, *p* < 0.001) in favor of FES-cycling. The pooled mean difference (random effect model) was −1.169 (Figure 3).

#### Subgroup Analysis and Meta-Regression for Training Sessions

**FIGURE 4:**
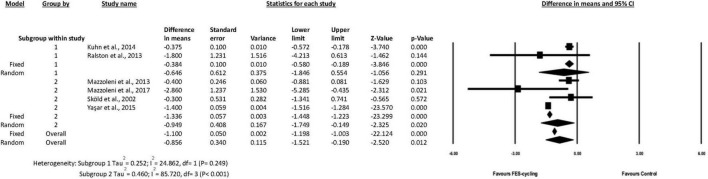
A forest plot of subgroup analysis for spasticity. Subgroup 1 was intervention sessions < 20 sessions, and subgroup 2 was ≧20 sessions.

The actual training sessions for subgroup 1 ranged from 8 to 12 (Ralston et al., 2013; Kuhn et al., 2014), whereas those for subgroup 2 ranged from 20 to 72 (Sköld et al., 2002; Mazzoleni et al., 2013, 2017; Yas̨ar et al., 2015). The results of subgroup analysis showed that MAS score decreased 0.949 significantly only in the subgroup 2 (95% *CI* = −1.749 to −0.149, *p* = 0.020, random effect model) (Figure 4). This suggested that above 20 intervention sessions of FES-cycling training were effective to improve spasticity.

**FIGURE 5:**
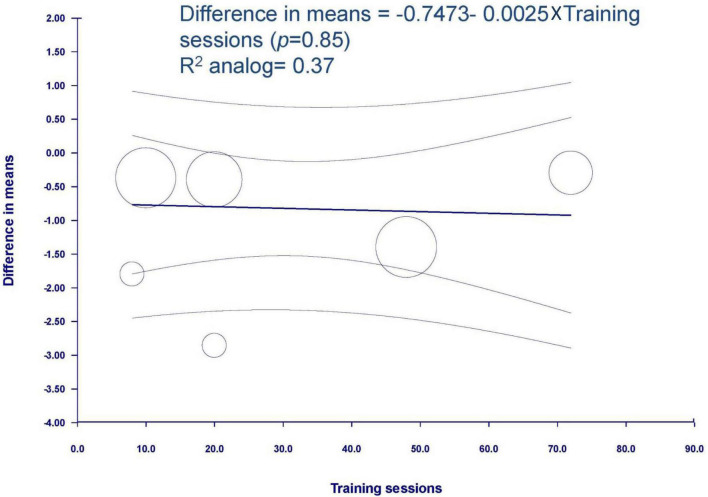
Meta-regression of spasticity on training sessions. Regression line and 95% *CI* were presented.

The result of meta-regression was not significantly associated between the training sessions and spasticity (coefficient = −0.0025, SE = 0.0129, *p* = 0.849, *R*^2^ analog = 0.37) (Figure 5).

#### Effects on 6 Min Walk Test and Timed Up and Go

**FIGURE 6:**
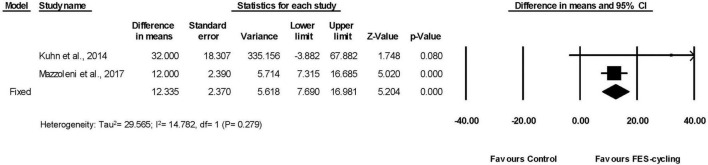
A forest plot of Six Min Walk Test (6MWT) following FES-cycling training.

Among all the included studies, two studies measured walking distance by 6MWT (Kuhn et al., 2014; Mazzoleni et al., 2017; Figure 6). The walking distance improved significantly (95% *CI* = 7.690–16.981, *p* < 0.001) in favor of FES-cycling training. The pooled mean difference (fixed effect model) was 12.335 m.

**FIGURE 7:**
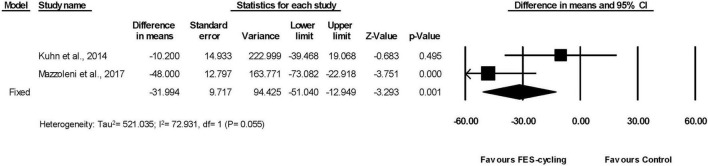
A forest plot of Timed Up and Go (TUG) after FES-cycling training.

These two studies had also measured TUG (Kuhn et al., 2014; Mazzoleni et al., 2017). The TUG significantly reduced (95% *CI* = −51.040 to −12.949, *p* = 0.001) in favor of FES-cycling training. The pooled mean difference (fixed effect model) decreased 31.994 s (Figure 7).

#### Effects on Lower Limbs Muscle Strength

**FIGURE 8:**
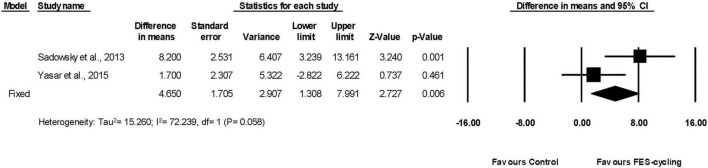
A forest plot of lower limbs muscle strength (LEMS) after FES-cycling training.

Two included studies had measured LEMS (Sadowsky et al., 2013; Yas̨ar et al., 2015). The pooled LEMS improved significantly (95% *CI* = 1.308–7.991, *p* = 0.006) after FES-cycling training. The pooled mean difference (fixed effect model) was 4.650 (Figure 8).

### Publication Bias

Supplementary Appendix 2 showed the funnel plot and Egger’s test of MAS. The funnel plot seemed asymmetrical. However, the Egger’s test showed no significant publication bias (*p* = 0.60094).

## Discussion

The current meta-analysis showed that FES-cycling training decreased spasticity, MAS score, in the individuals with SCI. The walking ability, 6MWT, TUG, and the strength of the lower limbs, LEMS, also increased. In additions, our meta-analysis showed that the number of training sessions was not linearly related to the decrease of spasticity. According to our meta-analysis, a threshold number of FES-cycling training sessions are 20 to obtain the efficacious decrease in spasticity.

### Decreasing Spasticity After Functional Electrical Stimulation-Cycling Training

The MAS is commonly assessed for spasticity in the clinic. The level of MAS represents the reflex and non-reflex components of spasticity (Chang et al., 2007). Abnormal spinal circuitry function is related to the reflex. H reflex reflects the excitation of the alpha motor neuron pool and is related to spasticity (Phadke et al., 2010). A previous study reported one session of sub-threshold electrical stimulation of gastrocnemius muscle (15–20 min, < 10 mA, 2,000–6,000 Hz) decreased Hmax/Mmax in healthy human adults (Yuan et al., 2010). Other study reported that one session of neuromuscular electrical stimulation of spastic quadriceps muscles (20 min, 300 μs, 25 Hz, and maximum 100 mV) reduced spasticity in people with SCI (Tancredo et al., 2013). Non-reflex part may come from the rhythmical leg movements. Repeated stretches of the ankle joint can reduce the resistance during passive stretch in the healthy adults (Avela et al., 1999) and H reflexes in individuals with SCI (Chang et al., 2007, 2013; Fang et al., 2015).

The effect of FES-cycling training on spasticity might partially come from the FES itself. Clinically, FES had been applied to improve spasticity. Sivaramakrishnan et al. (2018) reported that both 30 min, one session of FES and transcutaneous electrical nerve stimulation (TENS) decrease spasticity measured by MAS in acute to chronic SCI. The review from Naro et al. (2017) reported that FES 30 min two times a day, 5 days a week for 4 weeks can decrease spasticity in patients with SCI and stroke. FES itself has been applied to reduce spasticity not only in SCI (Bochkezanian et al., 2018) but also in stroke or cerebral palsy (Ho et al., 2014; Naro et al., 2017). A previous study also reported high-intensity knee extension NMES training two times a week for 12 weeks decreased spasticity in the patients with chronic SCI (Bochkezanian et al., 2018). However, a previous meta-analysis from Thomaz et al. (2019) reported that electrical stimulation did not improve spasticity in people with SCI. That could be due to their included studies were heterogeneous in intervention methods, such as FES walking, FES-cycling, and TENS. In our meta-analysis, we specifically selected FES-cycling training as the intervention to examine its effect on spasticity. The results showed that the effect of spasticity reducing was not simply from electrical stimulation.

The effect of reducing spasticity by FES-cycling training might also be associated with the cycling part. Cycling training was suggested to have effect on neural plasticity through the normalized spinal reflexes caudal to spinal injury level in the humans and rats (Lynskey et al., 2008). Cycling exercise could generate joint motions on the hips, knees, and ankles. The previous studies showed that passive cycling of ankle joint at a speed of 50 cycles/min decreased the reflex excitability in patients with SCI after one bout (Fang et al., 2015) and multiple sessions of training (Chang et al., 2013).

Several possible underlying mechanisms might be responsible for FES-cycling training to reduce spasticity. One is the restoration of post-activation depression. Post-activation depression is a pre-synaptic inhibition which serves to stable the joint and prevent clonus (Masakado et al., 2005; Yang et al., 2015). Post-activation depression was found to be decreased in the chronic phase after SCI that was considered as a cause of spasticity (Chang et al., 2013). A previous study showed that post activation depression could be restored after passive cycling training on ankle joints for 4 weeks with a parallel improvement of spasticity in the individuals with chronic SCI (Chang et al., 2013). Another possible mechanism for spasticity reduction might be through Renshaw cell inhibition and/or recurrent inhibition (Milosevic et al., 2019). The electrical current activated the muscles orthodromically and antidromic volley depolarized the α-motoneuron cell bodies. The antidromic impulse might also activate Renshaw cell interneurons resulting in recurrent inhibition (Mazzocchio et al., 1994). The other possible reason for reducing spasticity by FES-cycling training might be muscle fatigue induced by FES intervention (Krause et al., 2008).

The current meta-analysis showed the score of the MAS significantly reduced 0.86 after FES-cycling training in people with SCI. No previous study reported the minimal clinically important difference of MAS for people with SCI. The possible clinical impact in contrast to the minimal clinically important difference obtained from the patients with stroke, which was 0.73 (Chen et al., 2019), suggested that FES-cycling training induced spasticity decrement might be beneficial for people with SCI clinically.

### Effect of Functional Electrical Stimulation Protocol on Spasticity

A previous systematic review performed by Bekhet et al. (2019) suggested the frequency, pulse duration, and current amplitude of ES to be 20–30 Hz, 300–350 μs, and > 100 mA for reducing lower limb spasticity in people with subacute or chronic SCI. In our current meta-analysis, 6 of 12 included studies used the frequency within the range of 20–30 Hz (Sköld et al., 2002; Krause et al., 2008; Szecsi and Schiller, 2009; Kuhn et al., 2014; Yas̨ar et al., 2015; Fattal et al., 2018). One study used a higher frequency (100 Hz) (Sadowsky et al., 2013). For the pulse duration, five included studies used 250–350 μs (Sköld et al., 2002; Ralston et al., 2013; Kuhn et al., 2014; Yas̨ar et al., 2015; Fattal et al., 2018), whereas the other six studies used 500–600 μs (Krause et al., 2008; Szecsi and Schiller, 2009; Reichenfelser et al., 2012; Mazzoleni et al., 2013, 2017; Sadowsky et al., 2013), and two studies did not reported the frequency (Mazzoleni et al., 2013; Popovic-Maneski et al., 2018) or pulse duration (Popovic-Maneski et al., 2018). In general, the included studies used a stimulation frequency agree with Bekhet’s suggestion, but the pulse duration was varied. This might suggest that the frequency requirement is more restricted than pulse duration while performing FES-cycling training. According to the review of Marquez-Chin and Popovic, 20 Hz is the minimum frequency to produce tetanic contraction (Marquez-Chin and Popovic, 2020). Since FES-cycling needs the muscle to produce tetanic contraction, this could explain why the frequency used in all studies was above 20 Hz. Therefore, our current meta-analysis suggests FES at a frequency range from 20 to 30 Hz is suitable for FES-cycling training to improve spasticity. One of the selected study by Sadowsky et al. (2013) used a high frequency and longer pulse duration (100 Hz and 500 μs). This might induce high frequency fatigue (Behringer et al., 2016), but the muscle fatigue was not reported. It may be that NMES/FES can be delivered using longer pulse duration and higher frequency (1 ms of pulse widths, frequency ∼100 Hz) that are used conventionally to reduce contraction fatigability and enhance the benefits of NMES/FES-based programs for the neuromuscular and cardiovascular systems by generating contractions through spinal and possibly transcortical “central pathways” (Collins, 2007; Bergquist et al., 2011; Bastos et al., 2021).

### Influence of Training Sessions

A previous meta-analysis on the patients with stroke showed that 15–20 sessions of NMES significantly decreased spasticity (Stein et al., 2015). Prior study reported more than 10-week FES altered the neural circuit on the patients with SCI (Aziz et al., 2009). Our study is the first meta-analysis to investigate and reveal that 20 FES-cycling training sessions are required to reduce spasticity in the patients with SCI. Although the disease courses and rationale of SCI and stroke are different, the sessions required for electrical stimulation on decreasing spasticity are similar. Neural circuit adaptations, such as normalization of H reflex and post-activation depression, are suggested to be an underlying mechanism to reduce spasticity (Chang et al., 2013). Our meta-analysis also found that the number of training sessions and the effects on reducing spasticity are not linearly related. The training effect may approach a plateau as training sessions increase.

### Influence of American Spinal Injury Association Level and Other Factors

The ASIA level might be the other factor to influence the effect of FES-cycling training on spasticity. Most included studies recruited subjects with SCI with mixed ASIA levels. Hence, it was subgrouped to complete (ASIA A) and incomplete (ASIA B, C, and D) injury. The results were that spasticity significantly reduced in complete and incomplete SCI in favor of FES-cycling training. The pooled mean differences in complete and incomplete subgroup were −1.08 (95% *CI* = −1.281 to −0.878, *p* < 0.001) and −0.731 (95% *CI* = −1.393 to −0.070, *p* = 0.030), respectively. The forest plot was shown in Supplementary Appendix 3. Since no previous study reported the minimal clinically important difference of MAS for people with SCI, the possible clinical impact in contrast to the minimal clinically important difference obtained from the patients with stroke, which was 0.73 (Chen et al., 2019) might demonstrate beneficial effect of FES-cycling training for people with SCI. The results of both complete and incomplete SCI exceeded clinically important difference of spasticity after FES-cycling training, and the reduction of spasticity was more in complete SCI than in incomplete SCI. Although people with incomplete SCI can perform voluntary exercise without the aid of FES-cycling, they still are benefited from FES-cycling training on the reduction of spasticity.

The included studies did not analyze the results by the level of injury. Not every included study reported the definite injury level, such as Sköld et al. (2002), which showed C1 to T1 injury level, and Szecsi and Schiller (2009) which reported C7 to T12. For the time since injury, some included studies showed a wide range of time since injury (less than 4 weeks to 17 years), and some did not report this data (Mazzoleni et al., 2013, 2017). The results of MAS in every included study were not reported by each level of injury or time since injury. This makes the subgrouping analysis not possible.

For the session duration, all the included studies had a similar session duration which was from 20 to 60 min per session. This might be restricted by tolerance of the subjects. The frequency of FES of included studies was from 2 to 4 times a week in all the included studies. This follows the general training guidelines. Therefore, subgroup analyzation of the influence of session duration and/or frequency could not be done with the available studies.

### Walking Abilities (Six Min Walk Test and Timed Up and Go)

Six Minute Walk Test and TUG are related to the walking abilities. Our meta-analysis revealed that FES-cycling training improved the walking abilities after SCI. Spasticity reduction could be a factor causing this improvement as it plays a role in abnormal gait patterns after SCI (Krawetz and Nance, 1996). According to a study, the spasticity presented by thoracic SCI is the most obvious, and their knee excursion and knee angular velocity showed the greatest deviation among all the SCI groups (Krawetz and Nance, 1996). Guimaraes et al. (2016) suggested that subjects with traumatic spinal cord injury above T12 level were the best potential candidates for FES-cycling training. The subjects in our current meta-analysis were mostly thoracic and cervical injury level. Meanwhile, reduction of spasticity after FES-cycling training showed beneficial effect on the walking abilities measured by 6MWT and TUG in SCI.

Six Minute Walk Test is also an indicator of endurance (Bennell et al., 2011). Our meta-analysis supported that the individuals with SCI could be benefited from FES-cycling to increase endurance. TUG is a common test of functional mobility. It was shown to be correlated with muscle strength of the lower extremities, balance, and gait speed in elderly adults (Podsiadlo and Richardson, 1991; Chen and Chou, 2017). Our results revealed that LEMS improved in favor of FES-cycling training. The enhancement of LEMS after FES-cycling training can promote TUG performance.

### Lower Limbs Muscle Strength

Our current meta-analysis showed that the pooled LEMS improved significantly after FES-cycling training although the included studies were few. To compare the two included studies, more improvement of LEMS was found in Sadowsky et al. (2013) study than in Yas̨ar et al. (2015) study. The impairment level of the subjects was different between Sadowsky et al. (2013) and Yas̨ar et al. (2015) studies. Most subjects from Sadowsky et al. (2013) study were ASIA A level, and all the subjects but one from Yas̨ar et al. (2015) study were ASIA D. Both studies showed LEMS increased significantly after FES-cycling training. It is possible that FES-cycling is effective on improving the strength of lower limbs in the subjects with different ASIA classifications, but the improvement is more in subjects with ASIA A than with ASIA D.

A previous narrative review proposed FES-cycling training of 45–60 min, three times a week for at least 4 weeks to improve muscle strength in people with SCI (Rosley et al., 2019). It has also been shown resistance training of 18–27 weekly sets, completed over 2 weekly sessions for 6 weeks (total 12 sessions) increased muscle strength in trained people (Heaselgrave et al., 2019). The FES-cycling training protocols of two included studies (Sadowsky et al., 2013; Yas̨ar et al., 2015) showed muscle strength increase with at least 4 weeks training. The time since injury in these two included studies was 27–96 months. Motor recovery had been evidenced within 18 months after SCI (Krawetz and Nance, 1996; Guimaraes et al., 2016; Luo et al., 2020). Therefore, we can rule out spontaneous recovery of muscle strength. The effect of LEMS improvement arises from FES-cycling training. NMES and FES training have been reported to increase muscle mass, mitochondrial oxidative enzyme activities, plasma glucose level, and circulating insulin (de Freitas et al., 2018; Gorgey et al., 2019). However, detailed discussion of underlying mechanism is beyond the scope of this study.

### Limitations

This meta-analysis has some limitations. First, most included studies were not RCT studies. Future studies with high quality RCT would be suggested to provide stronger evidence. Second, MAS of different lower extremities joints were pooled for analysis. Future studies differentiating joint MAS are suggested for clinical management. Third, due to the design of included studies, factors, such as level of injury, time since injury, frequency of FES, or session duration could not be analyzed by subgrouping. Future studies are suggested to clarify the differential effects on level of injury, frequency of FES, and treatment session duration. Fourth, the total number of included studies was small. A meta-analysis is a statistical combination of results from two or more separate studies. Variation across studies (heterogeneity) is considered. The prediction intervals from the random-effect models are a useful device for presenting the extent of between-study variation (Deeks et al., 2021). Although the total number of studies was small, we chose the random-effect models to estimate the effect and made careful interpretation.

## Conclusion

Functional electrical stimulation-cycling training reduced spasticity in the individuals with SCI. The walking abilities and the strength of the lower limbs also improved following FES-cycling training. The current meta-analysis also showed that the number of training sessions was not linearly related to the decrease of spasticity. Instead, our meta-analysis suggested that 20 sessions of FES-cycling training are required to obtain the efficacy to decrease spasticity. FES-cycling training was a potentially beneficial rehabilitation strategy to improve spasticity in the clinics or at home for people with SCI.

## Data Availability Statement

The original contributions presented in the study are included in the article/Supplementary Material, further inquiries can be directed to the corresponding author/s.

## Author Contributions

C-YF and AS-YL performed the literature search and eliminated obviously irrelevant studies. C-YF, AS-YL, and Y-JC ranked the remaining studies as relevant, possibly relevant, and irrelevant according to our inclusion criteria, and performed the analyses. J-LT and H-CY checked inclusion of the probably relevant and relevant studies according to the PICOS scheme of each study. H-LC and R-SC extracted trial and outcome data from the trials. All authors revised it critically, interpreted the data, and read and approved the final manuscript.

## Conflict of Interest

The authors declare that the research was conducted in the absence of any commercial or financial relationships that could be construed as a potential conflict of interest.

## Publisher’s Note

All claims expressed in this article are solely those of the authors and do not necessarily represent those of their affiliated organizations, or those of the publisher, the editors and the reviewers. Any product that may be evaluated in this article, or claim that may be made by its manufacturer, is not guaranteed or endorsed by the publisher.

## Footnotes

1
https://www.crd.york.ac.uk/prospero/display_record.php?ID=CRD42021230762

## Abbreviations

SCI: spinal cord injury
FES-cycling: functional electrical stimulation cycling
NMES: neuromuscular electrical stimulation
AS: Ashworth Scale
MAS: Modified Ashworth Scale
6MWT: 6 Min Walk Test
TUG: Timed Up and Go
LEMS: lower limbs muscle strength
FES: functional electrical stimulation
TENS: transcutaneous electrical nerve stimulation
RCT: randomized control trial
CI: confidence interval
PAD: post activation depression.
