# Supplementary material for: The Effect and Dose-Response of Functional Electrical Stimulation Cycling Training on Spasticity in Individuals With Spinal Cord Injury: A Systematic Review With Meta-Analysis
Source: Front Physiol. 2021 Nov 19;12:756200. doi: 10.3389/fphys.2021.756200 (PMC8640241; doi:10.3389/fphys.2021.756200)

**Appendix 1** Searching strategy

**Databases:** PubMed, Scopus, Medline (Proquest), Embase and Cochrane Central Register of Controlled Trials [CENTRAL]

SCI related

1. Spinal cord injury
2. SCI
3. Spinal cord ischemia
4. Spinal cord vascular diseases
5. Spinal cord neoplasm
6. Spinal cord disease
7. Spinal cord compression
8. Spinal cord laceration
9. Myelopathy
10. Spinal tumor
11. Spinal damage
12. Spinal trauma
13. Spinal fracture

Cycling related

1. Cycling
2. Passive movement
3. Movement Therapy
4. Continuous Passive

Spasticity related

1. Spasticity
2. Hypertone
3. Spasm
4. Clonus
5. 1 or 2 or 3 or 4 or 5 or 6 or 7 or 8 or 9 or 10 or 11 or 12 or 13
6. 14 or 15 or 16 or 17
7. 18 or 19 or 20 or 21
8. 22 or 23 or 24 or 25
9. 22 and 23 and 24 and 25

**Appendix 2** Funnel plot of MAS standard error by difference in means and Egger’s test.


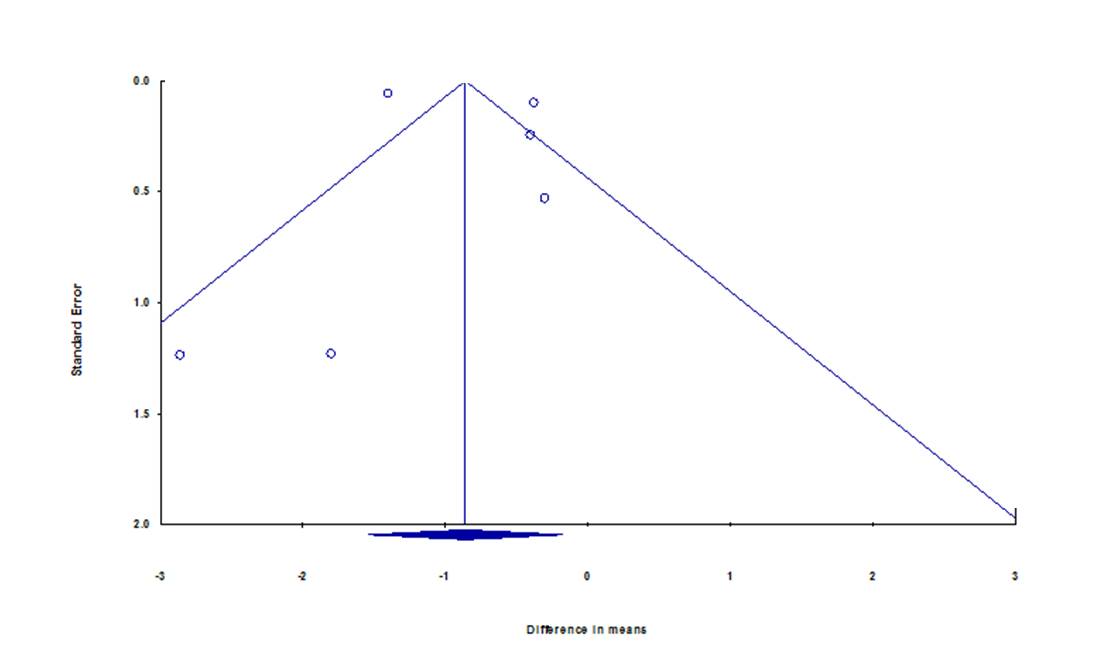


| Egger’s regression intercept | |
| --- | --- |
| Intercept | 1.47997 |
| Standard error | 2.60956 |
| 95% lower limit (2-tailed) | -5.76533 |
| 95% upper limit (2-tailed) | 8.72526 |
| t-value | 0.56713 |
| df | 4.00000 |
| P-value (2-tailed) | 0.60094 |

**Appendix 3** Forest plot of subgroup analysis for spasticity. Subgroups were complete (ASIA A) and incomplete (ASIA B, C, D) two groups.


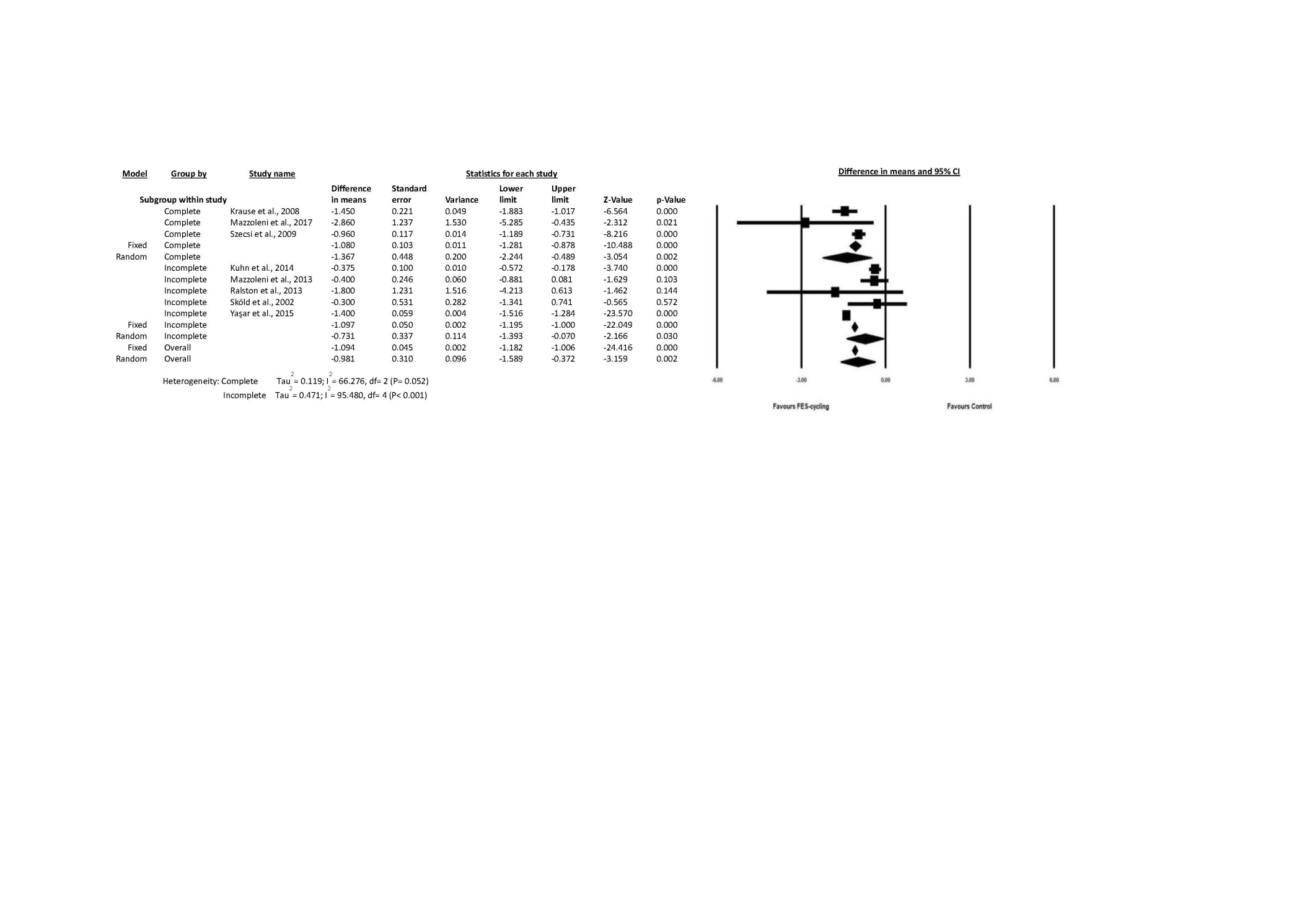

Supplement: Supplementary file 1 [file Data_Sheet_1.docx]
